# From the lab to the people: major challenges in the biological treatment of Down syndrome

**DOI:** 10.3934/Neuroscience.2021015

**Published:** 2021-02-09

**Authors:** Jean A Rondal

**Affiliations:** University of Liège, Belgium

**Keywords:** Down syndrome, trisomy 21, chromosome correction, induced pluripotent stem cells, neural stem cells, gene editing, CRISP-Cas9, epigenetic regulation, epigallocathechin-3-gallate

## Abstract

Down syndrome (DS) refers to a genetic condition due to the triplication of human chromosome 21. It is the most frequent autosomal trisomy. In recent years, experimental work has been conducted with the aim of removing or silencing the extra chromosome 21 (C21) in cells and normalizing genetic expression. This paper examines the feasibility of the move from laboratory studies to biologically treating “bone and flesh” people with DS. A chromosome or a gene therapy for humans is fraught with practical and ethical difficulties. To prevent DS completely, genome editing would have to be performed early on embryos in the womb. New in vitro findings point toward the possibility of epigenetic silencing the extra C21 in later embryonic or fetal life, or even postnatally for some aspects of neurogenesis. These possibilities are far beyond what is possible or allowed today. Another approach is through epigenetic regulation of the overexpression of particular genes in C21. Research with mouse modeling of DS is yielding promising results. Human applications have barely begun and are questioned on ethical grounds.

## 1. Introduction

DS is the most frequent autosomal trisomy (C21) occurring naturally approximately one time in 700 live births. Human C21, technically called Hsa21, contains about 225 protein-coding genes and between 165 to 404 non-coding RNAs (ribonucleic acid) regulating gene expression. Overproduction of proteins linked to gene triplication causes a series of anomalies involving heart, nervous system, gastro-intestinal tract, decreased volumes of brain frontal and temporal cortices, hippocampus, cerebellum, and brain stem. Anomalies of neural connectivity are also observed [1].

DS appears in several forms: (1) Standard trisomy 21-T21, (95% of the cases; karyotype 47 + 21); (2) Mosaic T21 (1 to 2% of the cases) where only a portion of the cells carries one extra Hsa21; (3) Robertsonian (centric fusion; nonreciprocal) translocations where participating chromosomes (pairs 13, 14, 15, 21, or 22) break at their centromeres and the long arms (q segments) fuse to form a single large chromosome with a single centromere. Robertsonian translocations involving C21 are: C21 with C21, C14, or more rarely C15: formulae 46 t (21; 21) + 21, 46 t (14; 21) + 21, and 46 t (15; 21) + 21, respectively. They account for 3% of the cases; (4) Partial T21 (less than 1% of the cases) result in only a segment of Hsa21 being triplicated [1].

DS maps to a region on the long arm of Hsa21 corresponding to band 21q22 [1]. A smaller region in Hsa21, labelled DSCR (Down Syndrome Critical Region), involving bands 21q21.1 and 21q22.2, includes about 50 protein-coding genes and a larger number of non-coding elements. This region arguably may harbor most of the critical determinants of the phenotype of the condition [2].

The cause of standard T21 is chromosomal nondisjunction mostly during meiosis I in the maternal egg. Paternal nondisjunction occurs during meiosis II in spermatogenesis. Translocations involving Hsa21 may occur de novo during syngamy or be inherited from parental genotypes (in about one quarter of the cases). Although parental genotypes have no in excess or deficit genetic material and their translocation is balanced, there is a 10–15% risk of having a child with DS if the mother carries the translocation and 2–3% risk if the father is the carrier [3].

Current work in cyto- and molecular genetics open the way for a biological treatment possibly conducive to a major improvement of the phenotype of persons with DS. The present paper identifies some of the major challenges in this vein of research. Some may seem insuperable in the present state of knowledge, technical know-how, biomedical ethics, and legal provisos. The reader is invited to consult a previous review article of mine [4] for further technical information on the studies motivating the following discussion. This paper is divided into three sections: (1) Epigenetic chromosome correction; (2) Deletion and epigenetic regulation of genes; (3) Ethical considerations.

## 2. Correcting extra Hsa21 in trisomic cells

Three publications have reported in vitro success in removing or silencing one extra Hsa21. Li et al. [5] generated induced pluripotent stem cells (iPSCs) from fibroblasts obtained from human adults with DS through genetic engineering using transcription factors [6]. They introduced a TKNEO fusion transgene carried by a modified adenovirus at the locus 21q21.3 of the gene *APP* (amyloid-bêta precursor protein) into one copy of Hsa21. This operation resulted in spontaneous loss of an entire copy of Hsa21 in a large majority of clones while point mutations, epigenetic silencing and TKNEO deletions occurred at lower frequencies. No damage to other chromosomes was evidenced. Disomic cells were observed to proliferate faster in co-cultures than trisomic counterparts.

Jiang et al. [7] reprogrammed fibroblasts obtained from human males with DS into iPSCs. They inserted a transgene XIST at locus 21q22 of the gene DYRK1A in one of the three Hsas21. Natural X dosage reduction in mammalian females is driven by a non-coding RNA, named XIST (for X-inactive specific transcript), produced from the inactive X chromosome. This RNA inactivates the deoxyribonucleic acid (DNA) of this chromosome through methylation and chromatin modification, turning it into a Barr body. Jiang et al.'s operation inactivated one of the three Hsas21 in 85% of the clones treated. Epigenetic silencing of a dozen genes on the inactivated Hsa21 was confirmed. No alteration of the other chromosomes was observed. A few sub-clones remained in the 245 colonies of cells treated showing either one Hsa21 fused with the XIST RNA, two Hsas21 in the same state, or the three Hsas21 fused with the XIST RNA. The resulting disomic cells exhibited a capacity for in vitro proliferation above trisomic counterparts.

Amano et al. [8] normalized the karyotypes in a culture of mouse embryonic stem cells engineered to become aneuploid or polyploid. They used a biologic made of a mammalian-specific gene, ZSCAN4 (for zinc finger and scan domain) containing 4 transcription factors, regularly expressed in preimplantation embryos. The biologics were encoded for delivery in a synthetic messenger RNA (mRNA) and Sendai virus vector (a murine retrovirus) and tested on iPSCs generated from fibroblasts obtained from human individuals with standard T21. After a few weeks, up to 24% and then 40% of cells with a normal karyotype emerged in the solution with no alteration of the remainder of the genome. The findings were confirmed by whole-exome sequencing.

It appears that in vitro correction of extra Hsa21 in trisomic cells is possible at no cost to the other chromosomes and that it restores a faster proliferation rate in the treated cells.

This is a remarkable achievement. Obviously, a number of steps lay ahead in the assessment of the innocuity and security of these techniques of genetic engineering. Empirical challenges are numerous. The next step is to test the applicability of the interventions to mouse models of T21. These models are most useful in genetic research even if DS in humans is orders-of-magnitude more complex. Genes in mice orthologous to Hsa21 are distributed and syntenically conserved on chromosomes 10 (39 genes), 16 (112 genes), and 17 (19 genes). For example, Ts65Dn mice have genes corresponding to 60% of the genes harbored by Hsa21, Dp(16)1/Yey mice duplicate a 23.3-megabyte-segment of Hsa21 (119 genes). They partially mimic the DS human condition including developmental delay, learning and memory deficits [9]. TcMAC21, a mouse model of DS carrying 93% of the protein coding genes on the long arm of Hsa21 has been created recently [http://dx.doi.org/10.7554/Elife.56223]. It recaptures many of the DS phenotypes including anomalies in heart, craniofacial skeleton, brain, cellular pathologies, synaptic impairment, learning and memory limitations. Mice with full T21, i.e., with all their genes ortholog to those on Hsa21, can be created by complex crossing of genetic lines. However, so far, they have proved difficult to produce, expansive, and short lived [10].

Interviewed by journalist Ian Sample (The Guardian international edition, Wed 17 Jul 2013), Jeanne Lawrence, head of the research team at the University of Massachusetts who published the article on “Translating dosage compensation to trisomy 21” [7], indicated that her team had started in vivo research to prevent DS in mice by silencing the extra chromosome 21 in early-stage embryos, which should correct the whole mouse. She acknowledged, however, that “it is not really practical in humans”.

There is more to the matter. In a paper just published, Czerminsky and Lawrence [11] report the results a new experiment demonstrating that contrary to prior belief epigenetic plasticity of DS iPSCs is retained at least for 35 days beyond the pluripotent stage. At this time, it is still strong enough to initiate chromosome-wide repression and correct T21 dosage in neural stem cells (NSCs) differentiating into neurons. This finding opens the way to epigenetic chromosome correction beyond the early stages of embryo development. Correcting a deficiency in the process of differentiation of trisomic neural stem cells into neurons appears to be possible by inducing XIST at different stages in neurogenesis. Dosage correction by XIST RNA promotes differentiation of NSCs into neurons by downregulating TTYHT1 expression (a gene located on chromosome 19, encoding a member of the Tweety family of proteins) and Notch pathway genes HEY1 (on chromosome 8) and RBP1 (on chromosome 3), a signaling pathway regulating cell differentiation.

Were do we stand in terms of possible application to humans?

Assuming that some time in the future it will become technically possible, safe, and legally authorized to correct all human cells with extra Hsa21 without harming the remainder of the genome. Such an intervention would have to be performed very early in embryonic life, optimally at the blastomere stage.

Embryo quality is routinely evaluated in the context of in vitro fertilization based on morphological and kinematic criteria. Embryo cleavage kinetics is assessed at day 1 (early cleavage, 25–27 hours after insemination of the oocyte), at day 2 (4 cells), and at day 3 (8 cells). Current imaging software facilitates the evaluation of embryo quality [12]. Embryo quality assessment can also be founded on genetic analysis as it is known that first gene expression occurs between the four- and eight-cell stages of embryonic development [13]. Preimplantation genetic tests are also performed for detecting aneuploidies. This requires a biopsy of the embryo aged 3 days for removing one or two cells. Increase in neonatal morbidity and mortality, as well as a genetic risk of chaotic embryo development, have been reported [14]. Day 5 or 6 trophectoderm biopsy is now recommended, as the result of the genetic tests are more accurate due to a better implantation potential of the embryo. Trophectoderm, as the name indicates, is the external cell layer of the embryo that will become the placenta when implanted into the woman's uterus [14].

A possible scenario for complete chromosome correction requires blastomere biopsy and genetic analysis to ascertain T21 before inserting the biologic agent able to normalize the aneuploidy and reimplanting the treated cell(s) into the embryo expecting cell proliferation to proceed normally from there on. The theoretical and technical knowledge needed for practicing such delicate maneuvers is not presently available. Binding legislations in most countries limit fundamental research on human embryos and embryonic stem cells as they involve destruction of the embryos. Clinically-oriented research is authorized only on supernumerary embryos from medically assisted procreation with the explicit consent of the donors. This is what motivated Yamanaka's team [6] to develop the technology of iPSC as a means of disposing of stem cells for experimental and clinical use without having to destroy embryos. And this is also why trophectoderm biopsy has become the preferred technique for evaluating the quality of the blastomeres in the context of in vitro fertilization [14].

In order to completely normalize embryonic development all of the 8 cells at day 3 post insemination would have to undergo chromosome correction. At this stage, embryonic cells are said to be multipotent. They no longer have the power to generate a complete organism but can differentiate into the various tissues. An intervention on fewer stem cells at this stage would induce a mosaicism of cells with the normal number of chromosomes and others with T21 in the growing organism. Chromosome correction at day 2 after insemination would not need to be performed on the 4 blastomeres as these earlier stem cells are totipotent, which means that each one is capable of generating a complete organism. However, whether performed at day 2 or 3 embryonic life, there would not be enough time between insemination and chromosome editing for rendering the therapeutic intervention practical.

The findings of the most recent study by the University of Massachusetts researchers who developed the chromosome correction XIST strategy [11] suggest the possibility of a new timing for XIST as a therapy for mitigating cell phenotype effects of DS. Many challenges remain, however. Not the easiest one is creating a small XIST transgene amenable to delivery methods. If authorized clinical procedures could be implemented warranting safe chromosome repression at the fetal or peri- and postnatal stages, this would provide a larger time window for intervention. Neurogenesis is largely completed before birth. However, important aspects of neural development continue quite some time after birth, such as myelinisation of the nerve fibers and synaptic pruning.

## 3. Deletion and epigenetic regulation of genes

Rather than repressing entire chromosomes, one can envisage removing or inactivating triplicated genes located on Hsa21. Efforts are being made to identifying the particular set of genes in DS whose overexpression are conducive to cognitive impairment.

A number of genes on Hsa21 show dosage effects in T21 that can be measured by biological activity (enzymatic, for example), quantity of proteins produced, or the amounts of RNAs [15]. They include DYRK1A (dual specificity tyrosine Y-regulation kinase 1A), APP (amyloid-beta precursor protein), and EURL (beta-catenin signaling modulator) that are involved in various cell functions and structural aspects of neurogenesis, OLIG1/2 (oligodendrocyte transcription) responsible for myelinating cells and oligodendrocyte differentiation, ERG (regulator of hemato-immune cells) and RCAN1 (calcineurin regulator) affecting the central nervous system. Overexpressed these genes are among the most noxious mechanisms in T21 brain etiopathology [16]–[19].

CRISPR-Cas9 (for clustered, regularly interspaced, short palindromic repeats, associated Cas 9 nuclease), dubbed the genetic scissors, has the capacity of inducing double strand DNA breaks. It can be used for DNA editing. However, this is forbidden in humans.

Even if safe, bioethically approved, and legally authorized, a CRISPR-Cas9 intervention in DS, involving a large number of dosage-sensitive genes, would be of a higher complexity than in many pathological conditions linked to a single gene alteration. However, it should still be possible to deal with individual genes in DSCR. Research with T21 mice models may prove instructive in the future [20].

Removing a particular gene from the DNA ribbon can also be performed using natural means. Chakrabarti et al. [17] eliminated one allele of each triplicated gene within the genome of Ts65Dn mice by breeding Ts65Dn females with OLIG1/2 double heterozygous males for normalizing the dosage of the genes OLIG1 and OLIG2 in the forebrain of the resulting pups. Returning Ts65Dn animals to disomy for OLIG1 and OLIG2 genes had the effect of normalizing neurogenesis.

Hsa21 harbors four major types of micro RNAs (miRNA-99a, miRNA-125b, miRNA-155, and miRNA-802). They are short non-coding RNAs mediating post-transcriptional gene silencing. Selective inactivation of some of these RNAs may be an efficient strategy for improving the DS phenotype. For example, Fillat et al. [21] employed an adenovirus-associated AAV2/1-shDYRK1A as a vector to carry a miRNA into the hippocampus of mice Ts65Dn for reducing the expressivity of the gene DYRK1A and in the control group a virus with a sequence that does not interfere with the gene DYRK1A (AAV2/1-scDyrk1A(SC). A statistically significant improvement was observed in the learning ability of the first group against the control one. However, experimental research with RNAs is not authorized in humans at the present time.

The epigenomics of DS has become an active area of research as epigenetics offers perhaps the best hope for biologically improving organic development in individuals with DS.

Epigallocathechin-3-gallate (EGCG), a polyphenol of green tea with antioxidant properties, is generating much interest. It is a natural inhibitor of the kinase encoded by the gene DYRK1A. A number of experiments have been conducted with mouse models for evaluating the efficiency of EGCG in rescuing various aspects of neurogenesis [22]–[24]. Administered in green tea extracts, it improves behavioral deficits in genetically modified or transgenic mice. In contrast, treatments with pure EGCG do not improve neurobehavioral phenotypes [http://dx.doi.org/1038/S41598-020-67133-Z]. The age of the animals at the time of treatment is a key variables. Studies carried out with adult mice show that learning and memory can be rescued to some extent but therapies administered early in brain development have more pronounced and durable effects [23].

Very few studies have been conducted so far for testing the behavioral effects of green tea extract containing EGCG in humans. De la Torre et al. [25] witnessed positive effects on visual recognition in a group of 29 adolescents and adults with DS, aged 14 to 29 years, following three months of daily treatment with EGCG green tea extracts, 9 mg per kilo of body weight, administered orally. No adverse event linked to the medication was observed. However, three months after the end of treatment, the participants' performance had returned to preintervention levels. In a second study, De la Torre et al. [26] tested the effect of a daily treatment with EGCG green tea extracts (with a dosage identical to the previous study), lasting for one year, coupled with a behavioral program of cognitive training.The sample included 84 adolescents and adults with DS (equal numbers of women and men), aged 16 to 34 years. They were divided into two groups: one treated with green tea extract contaning EGCG and undergoing cognitive training, the other receiving a placebo and the same cognitive training as the first group. Long-term administration of the medication in the experimental group was well tolerated. A battery of neuropsychological tests was administered at the end of the study. Participants treated with green tea extract containing EGCG exposed to cognitive training showed a statistically significant superiority in memory, in visual recognition, and in several adaptive tasks (e.g., daily routines abilities). A retest 16 months later showed partial persistence of the effects measured at the end of the intervention. It follows from these two experiments that green tea extract contaning EGCG alone does not induce lasting cognitive advantage in persons with DS whereas the combination of green tea extract containing EGCG and cognitive training brings about continuing cognitive benefits.

A possible caveat in the studies of De la Torre et al. is that the participants may have been already too advanced in age. Extrapolating from their own studies with murine models of T21, Stagni et al [23] suggest that treatment with green tea extract contanining EGCG in humans ought to start early, optimally during pregnancy. Green tea extract contaning EGCG administered to pregnant rats does not have teratogenic effects [27], but it is not known whether it might have adverse consequences on human pregnancies.

Overexpression of the gene APP is implicated in the “amyloid cascade”, related to the pathogenesis of Alzheimer disease (AD) affecting approximately 20% of persons with DS beyond 40 years of age, 40% beyond 50 years, 80% and more beyond 60 years [28]. Amyloid-beta peptides 40 and 42 accumulate in brain fluids and initiate the formation of extracellular plaques in the brain parenchyma. This accelerates the aggregation within brain neurons of a protein called TAU, naturally involved in cell microtubules, that becomes hyperphosphorylated and destroys the neuronal tissues [29]. Diffuse deposition of amyloid-beta proteins in brain tissues anticipating the accumulation of fibrillar amyloid starts around 35 years of age in DS [30].

It would make sense to target the APP gene and its peptide products in attempting to prevent an increase of cognitive difficulties with age in persons with DS. New molecules are needed devoid of side effects and capable of inhibiting APP overproduction or immunizing against amyloid-beta peptides accumulation in the intracellular domain. Researches on these topics with mouse models of DS are in the pipeline [31],[32].

Overexpression of gene DYRK1A is also thought to contribute to the hyperphosphorylation of the TAU protein leading to the neurofibrillation of neuronal bodies in AD [33],[34]. Attempts at reducing DYRK1A overexpression are also on the research agenda. For example, Garcia-Cerro et al. [35] have normalized DYRK1A dosage by breeding Ts65Dn mice with a triplication of this gene with mice trisomic for the same DNA segment but without the gene DYRK1A, producing Ts65Dn mice with normal dosage of this gene and a normal concentration of protein APP in cerebral cortex, hippocampus, and cerebellum. Kawakubo et al. [36] have treated fibroblasts obtained from persons with DS and AD pathology with harmine, a vegetal alcaloid antagonist of protein DYRK1A. Results show a decrease in the concentration of DYRK1A proteins in the fibroblasts. However, harmine is not a viable option to use in individuals with DS because of the risk of seizures and other disorders.

## 4. Ethical considerations

Inglis et al. [37] have distributed a questionnaire to members of the Lower Mainland Down Syndrome Society in British Columbia, Canada. It was completed by 101 parents. 41% responded that they would biologically treat their child of DS if it were possible. 27% said they would not biologically treat their child, and 32% were unsure. The major motivation for a treatment was to increase the child's autonomy.

An anonymous survey was conducted by Long et al. [38] with parents listed in the US Down Syndrome Registry (Eunice Kennedy Shriver National Institute of Child Health and Human Development) regarding the administration of green tea extracts containing EGCG. Parents who reported supplying their children with such extracts were younger, highly educated, and regularly consulted available scientific sources. The children receiving green tea extracts tended to be characterized as less severely disabled to begin with. Caregivers not giving green tea extracts to their children stated having doubts regarding treatment effectiveness and were concerned with the possible negative side effects.

Another survey, conducted by Riggan et al. [39] in the United States, recorded parents' opinions on hypothetical scenarios describing prenatal chromosome silencing and pediatric pharmacological intervention aiming at improving neurocognition in children with DS. A slim majority were being open to considering the availability of the therapies while expressing concern about the risks involved and the safety of pharmacotherapy. Some parents were also afraid of possible changes in their children's personality as a consequence of the treatments.

A group of researchers from the Department of Pediatrics, the Peabody College and the Divinity School joined with parents at Vanderbilt University, in Nashville, Tennessee, in April of 2011, to discuss ethical considerations of biologically treating disabilities. No minutes of the meeting were rendered public. However, Jennifer Wetzel posted a few extracts of the discussions on news.vanderbilt.edu (April 14, 2011). Robert Hodapp, Professor of special education and Director of research of the University Center for Excellence in Developmental Disabilities, commented that there was a palpable tension within the disability community between living with and biologically treating the disability. Parent advocate Sheila Moore, mother to a 21-year-old with Down syndrome and Executive director of the Down Syndrome Association of Middle Tennessee, said: “I wouldn't change [my son] for anything because without Down syndrome he wouldn't be the same person, and he has such beautiful, loving qualities. But, if I could cure his medical issues, help him find a good job, or do anything to make his quality of life better...”.

Not surprisingly parents are preoccupied with the efficacy and the safety of possible future attempts at improving the biological condition of their children with DS. However, considering the limited information that they have at disposal most of the time, it is interesting to note that almost half of them are not prejudiced against the possibility of a biological treatment. An argument of economic equity has been raised, however. The cost of chromosome or gene therapy is hypothetical as no gene therapy has been approved at this time. But it is likely to be expensive and only a select few might be able to afford it (The Bioethics Project, Ethics Institute at Kent Place School, Summit, New Jersey, 2016–2017, blogs.kentplace.org).

As indicated, some parents are afraid that biologically treating their children with DS would alter their beloved personality. Although deserving utmost respect, this opinion is based on what may appear as a forced dichotomy between respect and treatment [40]. The prospect in biologically treating persons with DS is not to modify their deeper inside but rather to try removing DS from within these persons as much as possible. There is no research evidence to date that suggests a person's personality would be altered. However, society's interaction with these persons could change and that might make a whole lot of difference.

## 5. Conclusions

Remarkable results have been reported in experimental works in vitro and with mouse models of DS. The question of their applicability to human individuals with DS is raised. It is important to distinguish between therapeutic intervention at the chromosome and at the gene levels. Correcting extra C21 in vitro is feasible and trials with mouse embryos are said to be in progress. To be complete, chromosome repression would have to be effectuated at the early-embryonic stage of development. At present, this is not authorized in humans nor is it technically possible. Even if it were, it would not be practical due to the insufficient time available between diagnosis and intervention with early embryos. New findings, however, may open the way to epigenetic chromosome correction beyond the early stages of embryo development.

Gene editing using CRISP-Cas9 is not allowed in humans and need to be tried with mouse models of DS. Reducing gene overexpression with natural products is authorized. Green tea extract containing EGCG has been used with mice with positive results. Corresponding attempts have been made with adolescents and adults with DS. They have met with mixed results. Recent research with mice suggests an earlier administration of green tea extract containing EGCG to children with DS in order to foster increased efficiency of the treatment.

Parents are divided on the subject of biological treatment for their children with DS. Ethical questions have begun to be raised. Some objections revolve around the belief that persons with DS are endowed with a particular personality that could be damaged in a therapeutic intervention.
